# Elevated Dkk1 Mediates Downregulation of the Canonical Wnt Pathway and Lysosomal Loss in an iPSC Model of Neuronopathic Gaucher Disease

**DOI:** 10.3390/biom10121630

**Published:** 2020-12-03

**Authors:** Manasa P. Srikanth, Ricardo A. Feldman

**Affiliations:** Department of Microbiology and Immunology, School of Medicine, University of Maryland, Baltimore, MD 21201, USA; MSrikanth@som.umaryland.edu

**Keywords:** Gaucher Disease, Wnt/β-catenin, Dkk1, Wnt3a, lysosomes, iPSC, neuronopathy

## Abstract

Gaucher Disease (GD), which is the most common lysosomal storage disorder, is caused by bi-allelic mutations in *GBA1*—a gene that encodes the lysosomal hydrolase β-glucocerebrosidase (GCase). The neuronopathic forms of GD (nGD) are characterized by severe neurological abnormalities that arise during gestation or early in infancy. Using GD-induced pluripotent stem cell (iPSC)-derived neuronal progenitor cells (NPCs), we have previously reported that neuronal cells have neurodevelopmental defects associated with the downregulation of canonical Wnt signaling. In this study, we report that GD NPCs display elevated levels of Dkk1, which is a secreted Wnt antagonist that prevents receptor activation. Dkk1 upregulation in mutant NPCs resulted in an increased degradation of β-catenin, and there was a concomitant reduction in lysosomal numbers. Consistent with these results, incubation of the mutant NPCs with recombinant Wnt3a (rWnt3a) was able to outcompete the excess Dkk1, increasing β-catenin levels and rescuing lysosomal numbers. Furthermore, the incubation of WT NPCs with recombinant Dkk1 (rDkk1) phenocopied the mutant phenotype, recapitulating the decrease in β-catenin levels and lysosomal depletion seen in nGD NPCs. This study provides evidence that downregulation of the Wnt/β-catenin pathway in nGD neuronal cells involves the upregulation of Dkk1. As Dkk1 is an extracellular Wnt antagonist, our results suggest that the deleterious effects of Wnt/β-catenin downregulation in nGD may be ameliorated by the prevention of Dkk1 binding to the Wnt co-receptor LRP6, pointing to Dkk1 as a potential therapeutic target for *GBA1*-associated neurodegeneration.

## 1. Introduction

Gaucher Disease (GD) is an autosomal recessive disease caused by monogenic mutations in the *GBA1* gene. *GBA1* encodes β-glucocerebrosidase (GCase), which is a 60 KDa lysosomal hydrolase that breaks down glucosylceramide (GlcCer). Mutations in *GBA1* affect GCase protein folding, resulting in endoplasmic reticulum retention and subsequent degradation [1,2]. The decreased enzyme activity in GD results in an accumulation of its lipid substrate GlcCer and its metabolite glucosylsphingosine in different organs, including the spleen, liver, bone marrow, and nervous system [3,4,5,6]. GD patients have varied onsets and severities of clinical manifestations, with a poor correlation between the genotype and phenotype [6,7]. Patients with neuronopathic subtypes (type 2 and type 3 GD) display severe neurological manifestations, including neurodegeneration, neuronal loss, ataxia, myoclonic seizures, and other abnormalities [8,9,10,11,12,13,14,15]. The rapid progression of the disease in type 2 GD (GD2) patients results in death within two years of age, while patients with type 3 GD (GD3) exhibit a slower clinical course [16,17,18]. Additionally, mutations in *GBA1* are the most common genetic risk factor for Parkinson’s disease (PD), with a 5–20 fold increased risk of developing PD in GD patients or carriers of *GBA1* mutations [19,20,21,22,23,24].

Neurodegeneration in neuronopathic GD (nGD) has been associated with dysfunction in the autophagy and lysosomal (ALP) pathway, which is essential for the survival of post-mitotic neurons [25,26]. Many studies have shown that *GBA1* mutations cause lysosomal abnormalities, defects in autophagic clearance, and the accumulation of protein aggregates in neurons [21,27,28,29]. Using an induced pluripotent stem cell (iPSC) model of GD, we have recently shown that ALP dysfunction was associated with a mammalian target of rapamycin (mTOR) hyperactivation, which resulted in transcription factor EB (TFEB) degradation [27,30]. TFEB is the master regulator of lysosomal biogenesis and autophagy [31,32]. Furthermore, Taelman et al. and others have shown a connection between the endo-lysosomal system and the Wnt/β-catenin pathway [33,34,35], suggesting that the ALP plays a role in the regulation of signal transduction networks essential for neuronal development.

The Wnt pathway is an important regulator of biological phenomena such as gastrulation, cell fate decisions, cell polarity, embryonic patterning, organogenesis, and the maintenance of stem cell pluripotency [36,37,38,39]. In addition, it also plays a vital role in early patterning of the central nervous system, as well as higher functions, such as memory, synaptic maintenance, and plasticity in the adult brain [40,41,42]. Due to the multi-faceted role of this signaling pathway in developmental and adult processes, its deregulation often leads to detrimental effects that have been linked to cancer, osteoporosis, and neurodegenerative disorders such as Alzheimer’s disease (AD) and PD [43,44,45,46,47]. The Wnt signaling pathway is highly regulated and complex, consisting of numerous *Wnt* genes, receptors, cofactors, and regulators. Among the different Wnt signaling mechanisms identified, the “canonical” Wnt pathway is the most studied and well-characterized. This signaling cascade begins with the interaction between Wnt ligands (secreted glycoproteins) and the receptor complex, consisting of Frizzled (Fz) seven-pass transmembrane receptor and single pass low density lipoprotein receptor-related protein 5/6 (LRP5/6) co-receptors [48]. This interaction facilitates the stabilization of β-catenin in the cytoplasm by preventing its association with the destruction complex consisting of glycogen synthase kinase 3β (GSK3β), casein kinase 1 alpha (CK1α), the adaptor axis inhibition protein (AXIN), and adenomatosis polyposis coli (APC). In particular, GSK3β directly controls β-catenin levels by phosphorylating it at three residues (Ser33, Ser37, and Thr41), which leads to its degradation by the proteasome [39,49,50]. Activation of the canonical Wnt pathway results in the sequestration of GSK3β into the endo-lysosomal compartment, thereby preventing the phosphorylation and degradation of β-catenin. The resulting accumulation of β-catenin in the cytoplasm triggers its translocation to the nucleus, where it modulates gene transcription by interacting with members of the T-cell factor/lymphoid enhancer-binding factor (TCF/LEF) class of transcription factors [48].

Wnt signaling can be modulated both intracellularly and extracellularly. Various extracellular antagonists of the canonical Wnt pathway have been identified, such as the secreted frizzled-related protein (sFRP), Wnt inhibitory factor 1 (Wif1), and Dickkopf (Dkk) family of secreted proteins. Dickkopf-1 (Dkk1), which is one of the four known Dkk family members, is a negative regulator of the Wnt pathway. Dkk1 inhibits Wnt signaling by binding to the co-receptor LRP6, thereby disrupting the LRP6-Fz complex and promoting LRP6 receptor internalization, hence limiting the availability of the receptor complex to Wnt ligands [51,52]. It has been hypothesized that Wnt antagonists such as Dkk1 could play a role in the pathogenesis of a number of diseases, making them important therapeutic targets [52,53]. For instance, Dkk1 has been implicated in AD [54]. Brains from AD patients and mouse models of AD display Amyloid beta (Aβ) and Tau aggregates, which induce the upregulation of Dkk1 [46,55]. Dkk1 is also elevated in GD; Lecourt et al. reported increased Dkk1 secretion by patient-derived mesenchymal stem cells [56], and Zancan et al. have shown increased *DKK1* gene expression in fibroblasts from type 1 GD patients [57]. However, a potential role of Dkk1 in nGD pathogenesis has not yet been explored.

Using GD patient-derived iPSCs, we previously showed that this in vitro system recapitulated pathological hallmarks of the disease in all of the cell types that we tested [58]. GD-macrophages have severe defects in clearing phagocytosed red blood cells, mimicking a pathological hallmark of GD [59]. Furthermore, GD-iPSC-derived hematopoietic progenitors showed decreased erythropoiesis and aberrant myelopoesis, reflecting cytopenias in GD patients [60]. Lastly GD-iPSC-derived neuronal cells display defects in the ALP and the downregulation of canonical Wnt signaling, contributing to neurodegeneration [27,30,61]. In this study, we utilized nGD iPSC-derived neuronal progenitor cells (NPCs) to investigate whether Dkk1 was involved in nGD pathogenesis. We found that nGD NPCs exhibit an elevation in Dkk1 mRNA and protein levels, which were responsible for suppressing activation of the Wnt co-receptor LRP6, increasing GSK3β activity, destabilizing β-catenin, and reducing lysosomal numbers. We also observed that the incubation of nGD NPCs with recombinant Wnt3a (rWnt3a) was able to override the effects of elevated Dkk1, showing that exogenous Wnt agonists are capable of reversing the phenotypic abnormalities of nGD neuronal cells. Our study reports, for the first time, increased Dkk1 expression in GD iPSC-derived NPCs, uncovers a novel link between elevated Dkk1 and deregulation of the lysosomal compartment, and suggests that Dkk1 may be a therapeutic target for nGD.

## 2. Materials and Methods

### 2.1. Generation of iPSC-Derived Neuronal Progenitor Cells (NPCs) 

The nGD and control (MJ) iPSC lines utilized in this study have been previously described [58,59]. The nGD iPSC lines were derived from one type 2 GD patient (GD2; genotype: W184R/D409H) and one type 3 GD patient (GD3; genotype: L444P/L444P). We used two different iPSC clones from each nGD patient. NPCs were derived from iPSCs as previously described [27]. Briefly, iPSCs cultured on irradiated mouse embryo fibroblasts (MEFs) were detached using 0.2% dispase and transferred to a 6-well ultra-low attachment plate (Costar) to generate embryoid bodies (EBs). The EBs were conditioned for neuronal induction through dual SMAD inhibition using 5 μM dorsomorphin (DM) and 10 μM SB431542 (SB) (Sigma–Aldrich, St. Louis, MO, USA). The iPSC-derived EBs were transferred to Petri dishes coated with Matrigel (Corning) and maintained in Dulbecco’s modified Eagle’s medium/F12 media (Life Technologies, Grand Island, NE, USA) containing 1X (*v*/*v*) N2 supplement (Life Technologies) and 20 ng/mL basic fibroblast growth factor (bFGF) (Peprotech, Rocky Hill, NJ, USA). The EBs adhered to the Matrigel and started the formation of neuronal rosettes within 7–10 days in culture. The rosettes were manually picked and expanded as NPCs in Neurobasal Medium (Life Technologies) containing 1X (*v*/*v*) GlutaMAX (Life Technologies), 1X (*v*/*v*) non-essential amino acids (Life Technologies), 1X (*v*/*v*) B27 supplement (Life Technologies), 1X (*v*/*v*) Pen/Strep, and 20 ng/mL bFGF (Peprotech), with a change of media every other day. The work with the human iPSC lines used in this study is considered non-human research; these iPSC lines are exempt under 45 CFR Part 46. This work was approved by the University of Maryland School of Medicine Institutional Review Board (IRB) on 15 July 2009 (HP-42545).

### 2.2. Chemical Reagents and Treatments 

rWnt3a (5036-WN) and rDkk1 (5439-DK) were purchased from R&D Systems (Minneapolis, MN, USA) and reconstituted as per the manufacturer’s instructions. Stocks of rWnt3a (200 μg/mL) and rDkk1 (100 μg/mL) were prepared in sterile PBS containing 0.2% bovine serum albumin. The final concentration used for both recombinant proteins was 100 ng/mL.

### 2.3. Quantitative PCR (qPCR)

To analyze gene expression in NPCs, mRNA was isolated using an RNA isolation kit (Qiagen, Germantown, MD, USA), and cDNA was synthesized using the iScript kit (Bio-Rad, Hercules, CA, USA). Utilizing SYBR Green PCR Master Mix (Thermo Fisher Scientific, Waltham, MA, USA), the gene expression was determined by qPCR (7500 Fast Real-time PCR System, Applied Biosystems, Foster City, CA, USA) in duplicate or triplicate wells. The mRNA expression for every gene was normalized to the corresponding values of *GAPDH* and the relative gene expression was calculated using the 2^(−ΔΔCt)^ method. The primer sequences used in this study were as follows:

*CYCD1*

Forward: 5′- CCG TCC ATG CGG AAG ATC- 3′

Reverse: 5′- GAA GAC CTC CTC CTC GCA CT- 3′

*AXIN2*

Forward: 5′- AGT GTG AGG TCC ACG GAA AC- 3′

Reverse: 5′- TGG CTG GTG CAA AGA CAT AG- 3′

*DKK1*

Forward: 5′- GAT CAT AGC ACC TTG GAT GGG- 3′

Reverse: 5′- GGC ACA GTC TGA TGA CCG G- 3′

*GAPDH*

Forward: 5′- CAA GAT CAT CAC GAA TGC CTC- 3′

Reverse: 5′- GCA TGG ACT GTG GTC ATG AGT C- 3′

### 2.4. Immunocytochemistry/Immunofluorescence

NPCs were plated in 8-well chamber slides (Thermo Fisher Scientific) and cultured as described above. When the slides were ready to be stained, media were aspirated from the chambers and the cells were washed once with Dulbecco’s Phosphate-Buffered Saline (DPBS) (Life Technologies, Waltham, MA, USA). The cells were then fixed with 4% (*v*/*v*) paraformaldehyde (Santa Cruz, Santa Cruz, CA, USA) for 15 min, washed thrice with DPBS, and blocked in Buffer A (PBS containing 8% FBS (*v*/*v*)) for 30 min at room temperature. Primary antibodies were prepared in Buffer B (Buffer A with 2 mg/mL saponin) and incubated for 1–2 h at room temperature or overnight at 4 °C. The cells were then incubated with the appropriate fluorochrome-conjugated secondary antibodies for 1 h at room temperature. Nuclei were labeled using 4′,6-diamidino-2-phenylindole (DAPI)-containing mounting medium (Vector Laboratories, H-1200, Burlingame, CA, USA).

Lysotracker staining was performed by adding 1 μM Lysotracker Red DND-99 (ThermoFisher Scientific) directly to cell culture medium and incubating it for 45 min to 1 h at 37 °C. The cells were then processed as described above. The following antibodies were used in this study: Primary antibodies; Cell Signaling Technology (Danvers, MA, USA): pLRP6 (Ser1490) (#2568), non-phospho (Active) β-catenin (#8814), and pGSK-3β (Ser9) (#9323); U. Iowa Developmental Hybridoma Bank (Iowa City, IA, USA): LAMP1 (H4A3); Santa Cruz: Dkk1 (sc-374574) and total β-catenin (sc-7199). Additionally, the secondary antibodies were Goat anti-rabbit Alexa Fluor 488 and Donkey anti-mouse Cy3 (Jackson ImmunoResearch Laboratories, West Grove, PA, USA).

### 2.5. Image Acquisition and Analysis

Confocal immunofluorescence images were taken using an inverted Nikon Eclipse Ti2 microscope attached to a spinning disk unit (CSU-W1, Yokogawa, Melville, NY, USA) and Hamamatsu sCMOS camera (Hamamatsu City, Japan). A Nikon oil immersion objective (Plan Fluor 40X, NA 1.30) was used for all imaging experiments. The excitation wavelengths used were 405, 488, and 561 nm for blue, green, and red fluorophores, respectively. Further image processing and analysis was conducted using Fiji software (version 2.1.0/1.53c, open source). The fluorescence intensity of the respective signal or Lysotracker counts was obtained from at least three independent replicates (3–5 different fields/replicate). The mean fluorescence intensity (MFI) and average puncti count were calculated accordingly.

### 2.6. Western Blot Analysis

NPCs were cultured in 12-well plates (Corning, Corning, NY, USA) and treated as indicated in the text. The cells were lysed directly in SDS sample buffer, sonicated, and denatured by heating at 95 °C for 5 min. The samples were then loaded onto 4–20% SDS/polyacrylamide gels (Bio-Rad, Hercules, CA, USA), and electrophoresis was performed at 100 V for 2 h. This was followed by the transfer of protein onto a nitrocellulose membrane, blocking with 5% milk in Tris-buffered saline Tween (TBS-T), and incubation with the indicated primary antibodies overnight at 4 °C. Anti-mouse or anti-rabbit HRP-conjugated secondary antibodies were added to the membrane for 1 h at room temperature. Membranes were developed with SuperSignal West Femto Maximum Sensitivity Substrate (ThermoFisher Scientific), and imaged using the Chemidoc system and Imagelab software (BioRad). Densitometry analysis was conducted using the Imagelab software (BioRad) and all proteins were normalized to β-actin.

### 2.7. Statistical Analysis

Data were analyzed using Prism software version 7.0a (GraphPad Software, San Diego, CA, USA). Significance was assessed using two-tailed unpaired Student’s *t*-tests for comparing two groups with a confidence interval of 95%. Results are expressed as the mean ± standard error of the mean (SEM).

## 3. Results

### 3.1. Upregulation of Dkk1 mRNA and Protein Levels in Neuronopathic GD NPCs

**Figure 1:**
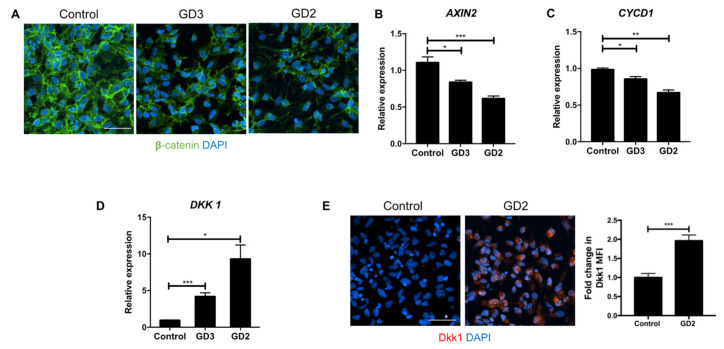
**Downregulation of canonical Wnt signaling with a concomitant elevation of Dkk1 in neuronopathic forms of Gaucher Disease (nGD) neuronal progenitor cells (NPCs).** (**A**) Representative confocal immunofluorescence images of control (WT) and nGD (GD2 and GD3) NPCs stained with antibodies to total β-catenin (green). Nuclei were stained with DAPI (blue). Scale bar: 50 μm. (**B**–**D**) qRT-PCR analysis of AXIN2, CYCD1, and DKK1 gene expression in control, GD2, and GD3 NPCs. The data are presented as the mean ± S.E.M (standard error of the mean). For AXIN2, *n* = 3 for control, *n* = 5 for GD2 (data were pooled from two clones derived from the same GD2 patient), and *n* = 3 for GD3 (data were pooled from two clones derived from the same GD3 patient). For CYCD1, *n* = 3 for control, *n* = 3 for GD2 (data pooled from two GD2 clones), and *n* = 2 for GD3 (data were obtained from one clone of GD3). For DKK1, *n* = 4 for control, *n* = 8 for GD2 (data were pooled from two GD2 clones), and *n* = 5 for GD3 (data were pooled from two GD3 clones). * *p* < 0.05, ** *p* < 0.01, and *** *p* < 0.001 between the indicated groups, as assessed by an unpaired Student’s *t*-test. (**E**) Representative immunofluorescence images of endogenous Dkk1 staining (red) in control and GD2 NPCs. Nuclei were stained with DAPI (blue). Scale bar: 50 μm. Quantitation of the mean fluorescence intensity (MFI) of Dkk1 is plotted to the right of the images. Results are expressed as the mean ± S.E.M (*n* = 3, *** *p* < 0.001, unpaired Student’s *t*-test).

We have previously shown that *GBA1* mutations downregulate the Wnt/β-catenin pathway in nGD iPSC-derived NPCs. In this system, the level of β-catenin, which is the main mediator of canonical Wnt signaling, was lower in nGD NPCs when compared to wild-type (WT) cells. This was attributed to the destabilization of β-catenin through proteasomal degradation since treatment with a proteasomal inhibitor was able to rescue the levels of both total and active (non-phosphorylated) β-catenin [61]. In accordance with our previous report, GD2 and GD3 NPCs exhibited reduced immunofluorescence staining of total β-catenin compared to control NPCs (Figure 1A). Additionally, when we analyzed the expression of two transcriptional targets of β-catenin, namely *AXIN2* and *CYCD1* by qRT-PCR analysis, there was a significant reduction in *AXIN2* and *CYCD1* transcript levels in the mutant NPCs, consistent with reduced β-catenin activity in these cells (Figure 1B,C).

We then investigated whether Dkk1 is involved in deregulation of the canonical Wnt pathway in nGD. To examine this question, we performed a qRT-PCR analysis of *DKK1* mRNA expression in WT and nGD NPCs. As shown in Figure 1D, both GD2 and GD3 NPCs displayed a significant elevation in *DKK1* expression relative to the control. Similarly, immunofluorescence staining revealed higher levels of Dkk1 protein in GD2 NPCs (Figure 1E). Taken together, we conclude that mutations in *GBA1* downregulate the Wnt/β-catenin pathway with concomitant upregulation of the Wnt antagonist Dkk1.

### 3.2. Recombinant Dkk1 Downregulates the Wnt/β-Catenin Pathway and Disrupts the Lysosomal Compartment in WT NPCs

**Figure 2:**
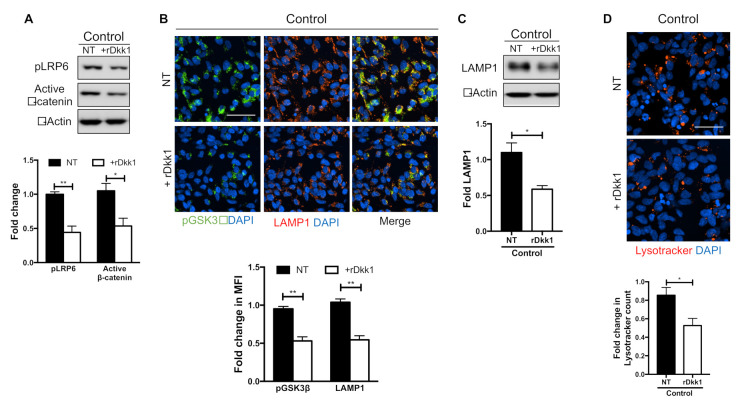
**Treatment of WT control NPCs with recombinant Dkk1 mimics the phenotypes observed in nGD NPCs.** (**A**) Western blot showing the expression of phosphorylated LRP6 (pLRP6) and (non-phospho) active β-catenin in control (WT) NPCs that were either left untreated (NT) or were treated with 100 ng/mL rDkk1 (+rDkk1) for 24 h. The proteins were normalized to β-actin, and fold-change was calculated with respect to NT WT. (**B**) Immunofluorescence staining of pGSK3β (Ser9) (green) and the lysosomal marker LAMP1 (red) in control NPCs that were either left untreated (NT, top panel) or were treated with rDkk1 (+rDkk1, bottom panel), as in A. The nuclei are labeled with DAPI (blue). Scale bar: 50 μm. The fold-change in mean fluorescence intensity (MFI) is plotted below the images. (**C**) Representative Western blot showing LAMP1 expression in control NPCs treated as in A. The bands were normalized to β-actin, and the plot below the WB shows fold-change. (**D**) Control cells were either left untreated (NT, top panel) or were treated with rDkk1 (+rDkk1, bottom panel). Untreated and treated cells were stained with Lysotracker (red) and imaged. Scale bar: 50 μm. The fold-change in Lysotracker count is plotted below the images. * *p* < 0.05 and ** *p* < 0.01 (*n* = 3, mean ± S.E.M, unpaired Student’s *t*-test).

Dkk1 is a Wnt antagonist that binds the Wnt co-receptor LRP6 and prevents formation of the LRP6-Fz receptor complex, thus suppressing the canonical Wnt pathway [52]. To investigate the mechanism of action of Dkk1, we treated control WT iPSC-derived NPCs with exogenous rDkk1 and analyzed the levels of various Wnt components. Firstly, incubation with rDkk1 decreased the activation of co-receptor LRP6, as shown by reduced levels of phosphorylated LRP6 (Ser1490) (pLRP6) (Figure 2A). As LRP6 is phosphorylated at several residues, including Ser1490, upon Wnt activation, our results suggest that rDkk1 was able to bind LRP6 and limit its availability to activating Wnt ligands. This was translated into the destabilization of β-catenin, as shown by lower levels of the non-phosphorylated, active form of β-catenin, following rDkk1 treatment (Figure 2A).

GSK3β is a constitutively active kinase that acts as a negative regulator of the Wnt pathway by phosphorylating β-catenin, causing its degradation by the proteasome. GSK3β activity is negatively regulated by phosphorylation at Ser 9 [49,62]. We previously reported that in nGD NPCs, there is a decrease in the levels of pGSK3β (Ser9), with no significant difference in the levels of total GSK3β [61]. To investigate whether rDkk1 had an effect on GSK3β activity, we examined the levels of pGSK3β (Ser9) in WT NPCs treated with rDkk1. As shown in Figure 2B, rDkk1 treatment lowered the levels of pGSK3β (Ser9) in WT NPCs, as determined by immunofluorescence staining. Therefore, treatment with rDkk1 recapitulated the decrease in levels of pGSK3β that we observed in nGD NPCs [61].

We and others have previously shown that the endo-lysosomal system positively modulates the canonical Wnt pathway by sequestering GSK3β into endo-lysosomal vesicles, thus stabilizing β-catenin levels [35,61,63,64]. We also showed that nGD NPCs exhibit severe lysosomal depletion and a reduced co-localization of pGSK3β with the lysosomal marker LAMP1 [61]. Hence, we wanted to determine whether rDkk1 treatment would also cause lysosomal depletion and alter the localization of pGSK3β. As shown in Figure 2B–D, rDkk1 treatment of WT NPCs resulted in deregulation of the lysosomal compartment. There were decreased levels of the lysosomal marker LAMP1, as determined by immunoblotting (Figure 2C) and immunofluorescence staining (Figure 2B), and a reduction in Lysotracker staining (Figure 2D). When we overlaid the pGSK3β signal with that of LAMP1 in untreated WT NPCs, there was almost complete co-localization of pGSK3β with LAMP1. On the other hand, after the incubation of WT NPCs with rDkk1, there was a considerable reduction in pGSK3β co-localization with LAMP1 (Figure 2B).

We can conclude from these results that the treatment of WT NPCs with exogenous Dkk1 was able to phenocopy the reduction in active β-catenin, lysosomal depletion, and alterations in the activity and subcellular distribution of GSK3β that we observed in nGD NPCs. These results lend strong support to the idea that Dkk1 is a key mediator of canonical Wnt downregulation in nGD neuronal cells.

### 3.3. Recombinant Wnt3a Treatment of nGD NPCs Rescues Wnt/β-Catenin Signaling and Restores the Lysosomal Compartments

**Figure 3:**
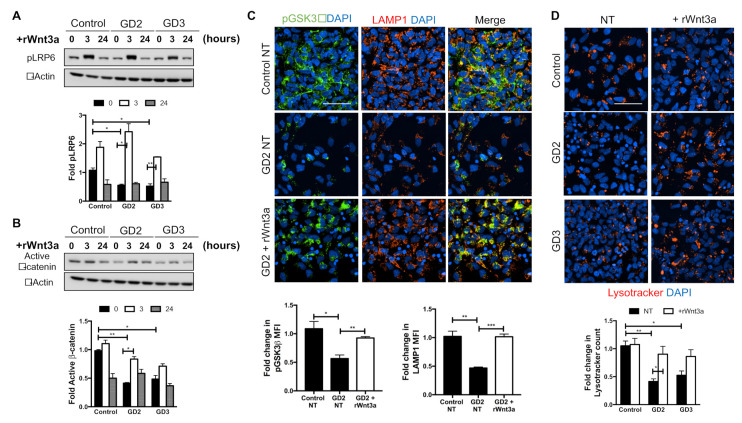
**Exogenous addition of recombinant Wnt3a reverses the abnormal phenotype of nGD NPCs.** (**A**,**B**) Western blots showing the levels of pLRP6 (**A**) and active β-catenin (**B**) in control, GD2, and GD3 NPCs treated with 100 ng/mL rWnt3a for 0, 3, and 24 h. The proteins were normalized to β-actin, and fold-change was calculated based on control (0 h). * *p* < 0.05 and ** *p* < 0.01 (*n* = 2, mean ± S.E.M, unpaired Student’s *t*-test between the indicated groups). (**C**) Immunofluorescence staining of pGSK3β (Ser9) (green) and LAMP1 (red) in untreated control NPCs and GD2 NPCs that were either left untreated (NT) or were treated with 100 ng/mL rWnt3a (+rWnt3a) for 3 h. Nuclei were stained with DAPI (blue). Scale bar: 50 μm. The fold-change in MFI is plotted below the images. * *p* < 0.05, ** *p* < 0.01, and *** *p* < 0.001 (*n* = 3, mean ± S.E.M, unpaired Student’s *t*-test). (**D**) Control, GD2, and GD3 NPCs were either left untreated (NT) or were incubated with rWnt3a as in B. The treated and untreated cells were stained with Lysotracker (red) and imaged. Nuclei were stained with DAPI (blue). Scale bar: 50 μm. The fold change in Lysotracker count is plotted below the images. * *p* < 0.05 and ** *p* < 0.01 (*n* = 4, mean ± S.E.M, unpaired Student’s *t*-test).

As our results implicated Dkk1 in suppression of the canonical Wnt pathway, we wanted to determine whether exogenous Wnt3a would be able to abrogate the effects of excess Dkk1 in nGD NPCs. To this end, we incubated control and mutant NPCs with rWnt3a for 3 or 24 h, and analyzed its effect on different components of the Wnt/β-catenin pathway. As shown in Figure 3A, immunoblot analysis demonstrated that rWnt3a triggered the engagement and activation of the receptor complex, as determined by an increase in pLRP6 levels. rWnt3a stimulated a robust response at 3 h, which returned to basal levels within 24 h. Similar results were obtained in GD2 and GD3 cells; under non-treated conditions, the mutant NPCs had significantly lower levels of pLRP6 when compared to WT NPCs, but rWnt3a was able to stabilize and increase pLRP6 levels. Importantly, rWnt3a treatment for 3 h also caused an increase in the levels of active β-catenin in GD2 and GD3 NPCs (Figure 3B), showing that this ligand was able to restore canonical Wnt signaling and overcome the inhibitory effect of elevated Dkk1 in the mutant NPCs.

We next investigated whether rWnt3a had any effect on pGSK3β (Ser9) and the lysosomal compartment. Figure 3C shows that the incubation of GD2 NPCs with rWnt3a for 3 h resulted in increased pGSK3β (Ser9) and LAMP1 staining, and lysosomal colocalization of these two proteins similar to control cells. Lysotracker staining also showed that rWnt3a prevented lysosomal depletion in GD2 and GD3 NPCs (Figure 3D). These results suggest that exogenous Wnt3a stabilized β-catenin through an increased sequestration of GSK3β into the endo-lysosomal compartment, and that lysosomal numbers also increased following treatment with the recombinant Wnt ligand. These results may suggest the existence of a bi-directional feedback mechanism between the canonical Wnt signaling network and the lysosome.

## 4. Discussion

In this study, we report that Dkk1 plays a key role in downregulation of the canonical Wnt pathway in nGD iPSC-derived NPCs, and that Wnt downregulation interferes with the lysosomal function. Dkk1 elevation in the mutant cells led to lysosomal depletion, reducing the sequestration of GSK3β into these organelles, thereby facilitating the phosphorylation and subsequent degradation of β-catenin. A critical role of Dkk1 in this process was confirmed by demonstrating that the addition of rDkk1 to WT NPCs recapitulated the mutant phenotype. Importantly, the phenotypic abnormalities of the nGD neuronal cells were reversed by incubation with rWnt3a. The addition of this ligand to mutant NPCs outcompeted the elevated levels of endogenous Dkk1, enabling GSK3β sequestration into the lysosomal compartment, protecting β-catenin from degradation, and preventing lysosomal depletion.

The Dkk family consists of four secreted proteins (Dkk1-4). Although Dkk2 has been shown to activate Wnt signaling, the other members of the family play mostly inhibitory roles [52]. Dkk1 was identified as a Wnt pathway antagonist that is essential for induction of the head in Xenopus during early embryogenesis [65]. Dkk1 exerts its action by interacting with the extracellular domains of Wnt co-receptors LRP5/6, in order to competitively inhibit the binding of Wnt1 and Wnt3 classes of ligands. Dkk1 deregulation has been implicated in human disease. Excess Dkk1 produced by myeloma cells suppresses osteoblast differentiation, resulting in lytic bone lesions in multiple myeloma [66,67]. Similarly, increased Dkk1 expression has been associated with the apoptosis of bone cells in femoral head osteonecrosis [68], with a low bone mineral density in children and adolescents with type 1 diabetes mellitus [69]. Additionally, elevated levels of Dkk1 have been observed in brains from AD patients and mouse models of AD and PD [45,46,47,55]. Furthermore, Dkk1 increases after NMDA excitotoxicity [70], and pharmacological inhibition of the NMDA receptor reduces the neurological manifestations in mouse models of nGD [71]. Therefore, Dkk1 elevation may play a wide role in neurodegeneration. The excess secretion of Dkk1 has also been reported in bone marrow-derived mesenchymal stem cells from GD patients [56], and Zancan et al. reported a higher *DKK1* mRNA expression in type 1 GD patient fibroblasts [57]. It will be interesting to determine whether Dkk1 is also elevated in nGD patient brains, and if there are increased levels of Dkk1 in serum or cerebrospinal fluid (CSF), which might then suggest the consideration of Dkk1 as a potential marker for nGD.

We have previously shown that in nGD NPCs, a loss of GCase activity downregulates the canonical Wnt pathway, leading to an increased degradation of β-catenin [61]. This was attributed to reduced lysosomal sequestration of GSK3β, which resulted in increased phosphorylation of β-catenin, thereby reducing its stability [61]. In the present study, we found that these alterations were likely mediated by an increased production of the Wnt antagonist Dkk1. Not only was Dkk1 elevated in the mutant NPCs, but the treatment of WT NPCs with exogenous rDkk1 resulted in diminished Wnt signaling due to interference with ligand binding to the LRP6 co-receptor. Remarkably, rDkk1 treatment also phenocopied the lysosomal depletion we observed in nGD cells (Figure 2). Therefore, Dkk1 appears to be an important mediator of the phenotypic abnormalities caused by GCase deficiency.

When nGD NPCs were treated with rWnt3a, the mutant phenotype was reversed, showing that this extracellular ligand was capable of overcoming the inhibitory effects of elevated, endogenous Dkk1. There was an increased activation of LRP6, β-catenin stabilization through GSK3β lysosomal sequestration, and an increase in lysosome numbers. The strong link between the Wnt and ALP pathways we observed suggests the existence of a bi-directional feedback loop, so that alterations in one pathway may affect the other. This idea is in line with other reports on the ability of the endo-lysosomal system to modulate Wnt signaling and in turn, the Wnt pathway regulating the lysosomal compartment [35,61,63,72,73,74,75]. Albrecht et al. reported an increase in both endocytosis and the lysosomal degradation of extracellular proteins within minutes of the addition of Wnt3a in NIH-3T3 fibroblasts. Additionally, Wnt3a treatment or inhibition of GSK3β in HeLa and HCC Alexander cells increased Lysotracker staining, and Cathepsin D and GCase activity [75]. A neuroprotective effect of Wnt ligands in vivo has also been reported [76,77,78,79,80]. The intranasal delivery of rWnt3a has been shown to modulate autophagy and regenerative pathways in a traumatic brain injury mouse model [79], and to have anti-apoptotic effects in a rat model of stroke [80]. Future studies will determine whether modulating the canonical Wnt pathway can help to ameliorate or prevent nGD neuronopathy.

As Wnt agonists (e.g., Wnt3a) and antagonists (e.g., Dkk1 and Sclerostin) act extracellularly, these are important pharmacological targets. For instance, the inhibition of Dkk1 and sclerostin using a bi-specific antibody has been shown to stimulate bone formation, increase the bone mass density and strength, and improve fracture healing in pre-clinical models of bone disease [81]. Similarly, a Dkk1 neutralizing antibody (BHQ880A, Novartis) has shown striking effects in multiple myeloma by increasing bone formation and inhibiting tumor growth both in vitro and in vivo [82,83]. Additionally, there are currently three ongoing clinical trials using a Dkk1-neutralizing monoclonal antibody (DKN-01, Leap therapeutics) for advanced biliary tract cancer (NCT04057365), prostate cancer (NCT03837353), and locally advanced or metastatic gastric or gastroesophageal junction adenocarcinoma (NCT04363801). Similarly, NCI8642 is a small molecule that was designed to displace Dkk1 from LRP5/6 and block the inhibitory effect of Dkk1 on Wnt signaling [84]. Derivatives of NCI8642 were found to lower Dkk1-induced Tau phosphorylation in SH-SY5Y cells [85]. Future studies will determine whether Wnt modulators have therapeutic potential to treat GD abnormalities, including the Wnt-related neurodevelopmental defects we reported in nGD [61] and bone abnormalities caused by defective Wnt signaling [57,86].

In conclusion, this study suggests that the elevation of Dkk1 observed in nGD NPCs is responsible for downregulation of the canonical Wnt pathway and deregulation of the lysosomal compartment. We also found that exogenous rWnt3a was able to outcompete the deleterious effects of elevated Dkk1 in the mutant cells. This study implicates Dkk1 in *GBA1*-associated neuropathology, and suggests that the canonical Wnt pathway is a potential therapeutic target for *GBA1*-associated neurodegeneration.
